# Hypoxia during incubation does not affect aerobic performance or haematology of Atlantic salmon (*Salmo salar*) when re-exposed in later life

**DOI:** 10.1093/conphys/coz088

**Published:** 2019-11-27

**Authors:** Andrew T Wood, Sarah J Andrewartha, Nicholas G Elliott, Peter B Frappell, Timothy D Clark

**Affiliations:** 1 Commonwealth Scientific and Industrial Research Organisation, Agriculture and Food, 3-4 Castray Esplanade, Battery Point, Tasmania 7004, Australia; 2 Tasmanian Institute of Agriculture, University of Tasmania, Private Bag 98, Hobart, 7001, Australia; 3 Institute for Marine and Antarctic Studies, University of Tasmania, 20 Castray Esplanade, Battery Point, Tasmania 7004, Australia; 4 School of Life and Environmental Sciences, Deakin University, Geelong, Victoria 3216, Australia

**Keywords:** hypoxia, Atlantic salmon, aerobic capacity

## Abstract

Hypoxia in aquatic ecosystems is becoming increasingly prevalent, potentially reducing fish performance and survival by limiting the oxygen available for aerobic activities. Hypoxia is a challenge for conserving and managing fish populations and demands a better understanding of the short- and long-term impacts of hypoxic environments on fish performance. Fish acclimate to hypoxia via a variety of short- and long-term physiological modifications in an attempt to maintain aerobic performance. In particular, hypoxia exposure during early development may result in enduring cardio-respiratory modifications that affect future hypoxia acclimation capacity, yet this possibility remains poorly investigated. We incubated Atlantic salmon (*Salmo salar*) in normoxia (~100% dissolved oxygen [DO, as percent air saturation]), moderate hypoxia (~63% DO) or cyclical hypoxia (100–25% DO daily) from fertilization until 113 days post-fertilization prior to rearing all groups in normoxia for a further 8 months. At ~11 months of age, subsets of each group were acclimated to hypoxia (50% DO) for up to 44 days prior to haematology, aerobic metabolic rate and hypoxia tolerance measurements. Hypoxia exposure during incubation (fertilization to 113 days post-fertilization) did not affect the haematology, aerobic performance or hypoxia tolerance of juvenile salmon in later life. Juveniles acclimated to hypoxia increased maximum aerobic metabolic rate and aerobic scope by ~23 and ~52%, respectively, when measured at 50% DO but not at 100% DO. Hypoxia-incubated juveniles also increased haematocrit and haemoglobin concentration but did not affect acute hypoxia tolerance (critical oxygen level and DO at LOE). Thus, while Atlantic salmon possess a considerable capacity to physiologically acclimate to hypoxia by improving aerobic performance in low oxygen conditions, we found no evidence that this capacity is influenced by early-life hypoxia exposure.

## Introduction

Aquatic hypoxia is increasing in prevalence throughout marine and freshwater systems, largely due to nutrient enrichment from anthropogenic sources (Diaz and Rosenberg, 2008; Diaz and Breitburg, 2009; Jenny *et al.*, 2016). The majority of aquatic organisms, including most fishes, are obligate water-breathers and therefore must endure or escape areas with low dissolved oxygen (DO). Salmonids may experience transient oxygen levels below 20% DO (% air saturation) during embryonic and larval development in under-gravel redds due to variations in water flow rates, embryo density and developmental stage (Ingendahl, 2001; Ciuhandu *et al.*, 2007; Greig *et al.*, 2007; Miller *et al.*, 2008; Dhiyebi *et al.*, 2013). Once free-swimming, salmonids may again encounter hypoxia in freshwater lakes and marine ecosystems due to expanding hypoxic zones and decreasing global aquatic oxygen content (Diaz and Rosenberg, 2008; Keeling *et al.*, 2010; Jenny *et al.*, 2016, Schmidtko *et al.*, 2017). Given the critical role of oxygen in driving performance and fitness, there is a key role to be played by conservation physiologists in helping to understand the responses and limits of aquatic organisms in low DO environments.

Oxygen uptake rate (aerobic metabolic rate, *Ṁ*O_2_) and critical oxygen level (O_2crit_) measurements are useful tools for assessing the extent to which aquatic animals can endure low DO environments. Energy-demanding activities such as digestion and swimming are dependent on the capacity for the oxygen uptake rate to increase beyond baseline levels (aerobic scope: the difference between minimum metabolic rate, *Ṁ*O_2min_ and maximum metabolic rate, *Ṁ*O_2max_) (Fry, 1971; Clark *et al.*, 2013; Claireaux and Chabot, 2016). The potential for fish to undertake oxygen-demanding processes decreases as oxygen availability declines (hypoxia), due to decreases in *Ṁ*O_2max_ and aerobic scope. Under severe hypoxia, the environmental oxygen concentration can decline below O_2crit_ (the DO below which *Ṁ*O_2min_ can no longer be maintained) and fish must either decrease their metabolic energy requirements or meet energy demands via anaerobic metabolism to maintain physiological homeostasis (Richards, 2009). If DO remains below O_2crit_, then loss of equilibrium (LOE) and death can ensue (Claireaux and Chabot, 2016). Thus, the aerobic scope and O_2crit_ of individuals provides insight into fish performance in low DO environments. Furthermore, the acclimation capacity of these traits may play some role in predicting population-level responses to increasingly hypoxic environments.

Fish typically physiologically acclimate to hypoxia by increasing oxygen uptake capacity, thereby improving hypoxia tolerance and aerobic performance. Fish immediately respond to hypoxia by releasing red blood cells via splenic contraction, followed by erythropoietin-induced red blood cell formation during prolonged hypoxia exposure (Boutilier *et al.*, 1988; Gallaugher and Farrell, 1998; Lai *et al.*, 2006; Tervonen *et al.*, 2006). As a result, the blood-oxygen-carrying capacity of fish increases during hypoxia exposure through increases in haematocrit (via increased number of red blood cells) and haemoglobin concentration ([Hb]). Improvements in blood-oxygen-carrying capacity can be associated with improvements in hypoxia tolerance. For instance, increased blood-oxygen-carrying capacity via increased haematocrit and [Hb] during hypoxia exposure is associated with a reduction in O_2crit_ and DO at LOE in killifish (*Fundulus heteroclitus*) (Borowiec *et al.*, 2015). Increases in acute hypoxia tolerance following hypoxia acclimation are also reported in Atlantic salmon (*Salmo salar*; decreased DO at LOE), zebrafish (*Danio rerio*; increased time to death at low DO) and brook trout (*Salvelinus fontinalis*; increased time to death at low DO) (Shepard, 1955; Rees *et al.*, 2001; Anttila *et al.*, 2015). However, physiological responses to hypoxia acclimation are not consistent across all studies. Hypoxia acclimation of rainbow trout (*Oncorhynchus mykiss*) does not affect maximum sustainable swimming speed (U_crit_), haematocrit or [Hb] despite increased blood-O_2_ affinity and decreased red blood cell ATP (Bushnell *et al.*, 1984). Similarly, haematocrit and [Hb] of steelhead trout (*O. mykiss*) is not affected by >17 weeks of acclimation to 40% DO (Motyka *et al.*, 2017). Thus, there may be species- and environment-dependent factors affecting physiological responses to hypoxia acclimation that have not been clearly elucidated.

It is possible that some of the disagreement between studies of hypoxia tolerance in fish stems from developmental plasticity, which is the ability of an individual to develop a range of phenotypes depending on its incubation environment. The resulting phenotype that develops in response to environmental conditions during early development may have positive or negative long-term consequences for fitness by altering an organism’s developmental trajectory (Beldade *et al.*, 2011; Burggren and Reyna, 2011; Gilbert, 2012; Bateson *et al.*, 2014). For example, hypoxia-incubated rainbow trout have a lower U_crit_ than normoxia-incubated trout after both groups have been subsequently held for 48 days in normoxia (Johnston *et al.*, 2013). However, acute hypoxia tolerance (DO at LOE) in Atlantic salmon and European seabass (*Dicentrarchus labrax*) exposed to developmental hypoxia is unaffected compared with normoxia-incubated individuals after more than 6 months in normoxia (Vanderplancke *et al.*, 2015; Wood *et al.*, 2017). Additionally, *Ṁ*O_2min_ is unaffected in zebrafish after 6 months in normoxia following developmental hypoxia, and *Ṁ*O_2min_, *Ṁ*O_2max_ and aerobic scope of Atlantic salmon and European seabass are unaffected after >6 months in normoxia following post-developmental hypoxia (Robertson *et al.*, 2014; Wood *et al.*, 2017; Zambonino-Infante *et al.*, 2017). While the long-term effects of early developmental hypoxia may not be apparent in environmentally benign conditions, it is plausible that developmental hypoxia may instead affect the animal’s future capacity to acclimate to changing DO environments (Beaman *et al.*, 2016). Indeed, temperature during early development can determine the long-term acclimation capacity of U_crit_ in zebrafish (*Danio rerio*), and *Ṁ*O_2min_ and aerobic scope in mosquitofish (*Gambusia holbrooki*) (Scott and Johnston, 2012; Seebacher *et al.*, 2014). Therefore, it is possible that developmental hypoxia could similarly influence the performance of individuals when re-encountering those conditions in later life.

**Figure 1:**
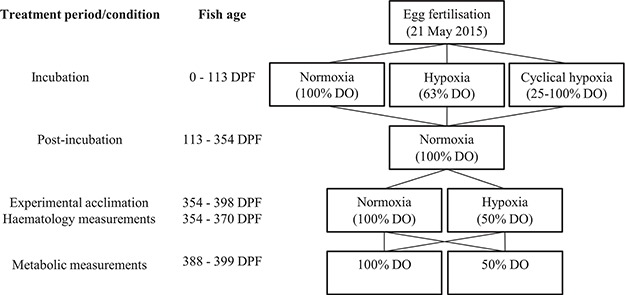
Experimental and measurement oxygen exposures and timing

Here, we measured haematology, aerobic capacity and hypoxia tolerance to compare the hypoxia acclimation capacity of juvenile Atlantic salmon following exposure to either normoxia (~100% DO), cyclical hypoxia (100–25% DO daily) or constant hypoxia (~63% DO) during early development. We hypothesized that early developmental hypoxia will increase hypoxia acclimation capacity later in life via larger and more rapid physiological responses upon re-exposure to hypoxia. These responses may include more pronounced increases in blood-oxygen-carrying capacity via increased haematocrit and [Hb], giving rise to increases in *Ṁ*O_2max_, aerobic scope and hypoxia tolerance (O_2crit_ and DO at LOE).

## Materials and methods

### Developmental incubation

Atlantic salmon were sourced from an established aquaculture selective breeding program in Tasmania, Australia, with founder stocks originating from the east coast of Canada in the 1960s (Elliott and Kube, 2009). On 21 May 2015, an equal proportion of all-female Atlantic salmon embryos were obtained from 16 families. The families were created by fertilizing the eggs from true female salmon with the milt from sex-reversed female (neo-male) salmon reared in freshwater at SALTAS, Wayatinah, Tasmania, Australia. The fertilized eggs were transported in freshwater to the CSIRO Hobart laboratories within 4 h of fertilization and randomly allocated between six Heath trays (L × W × H = 39 × 32 × 5.5 cm; MariSource, USA) at a density of 2400–2800 eggs per tray and maintained at ~8°C.

Embryos and alevins (yolk-sac larvae) were exposed from fertilization until 113 days post-fertilization (DPF; ~ 910 degree days) to one of three treatments (two replicate Heath trays per treatment): 99.6 ± 1.6% DO at 8.05 ± 0.20°C (normoxia; mean ± S.D.), 63.0 ± 3.3% DO at 8.03 ± 0.16°C (hypoxia) or 24-h cyclical hypoxia between 100 and 25% DO (66.0 ± 22.7% DO; 8.12 ± 0.19°C; Figs 1 and S1). The cyclical hypoxia treatment spent ~2 h per day both below 30% DO and above 95% DO, with DO increasing or decreasing between daily maximum and minimum levels at ~6.5% DO h^−1^. The Heath tray system was modified to supply each tray individually with water at 10 L min^−1^. The hypoxia and cyclical hypoxia groups received water from separate 200-L treatment sumps that exchanged water with a 600-L semi-closed recirculating filtration system (Tropical Marine Centre, UK). Water was supplied to the normoxia group directly from the latter-mentioned 600-L recirculating filtration system. DO was maintained at hypoxia treatment levels within the 200-L sumps by nitrogen injection and at 100% DO within the recirculating filtration system by oxygen injection. The gas injection was controlled by an OxyGuard Pacific oxygen monitoring system (OxyGuard International, Denmark) and PowerLab 4SP (ADInstruments, Australia). Water temperature and room air temperature were maintained at ~ 8°C by a heat exchanger system controlled by a central building management system (Building Automation Controls, Tasmania, Australia).

Fungal growth was prevented by treating the eggs with formalin (39% w/v formaldehyde) at 1.5 mL L^−1^ for 15 min on five occasions between 8 and 33 DPF according to industry best practices. The eggs were shocked once by physical agitation between 40–42 DPF to assist removal of dead eggs by turning them white, and dead embryos and alevins were removed as necessary from 40 to 113 DPF.

### Post-incubation rearing

At 113 DPF, ~ 600 alevins were transferred from each Heath tray to ~ 42-L glass aquaria (L × W ×H = 44 × 30 × 30 cm or 60 × 24 × 30 cm) containing normoxic freshwater supplied from a 600-L semi-closed recirculating filtration system (Tropical Marine Centre, UK). Each replicate Heath tray was transferred to an individual tank, resulting in two replicate tanks per developmental treatment group (six tanks in total). Water temperature was increased from 8 to 10°C over a 2-h period and DO increased to 90–100% from incubation treatment levels (cyclical hypoxia treatment already >90% DO at transfer). All tanks were fed daily with an equal ration of commercial Atlantic salmon pellet feed (Skretting, Tasmania, Australia). Fish were maintained under these conditions for ~33 weeks, and stocking density throughout rearing was maintained below 18 g L^−1^ by periodically culling haphazardly selected individuals.

### Experimental acclimations

At 342 DPF, the fish were distributed between two six-tank freshwater aquarium systems, maintaining two replicate tanks per developmental treatment within each aquarium system as during post-incubation rearing (Fig. 1). The fish were initially stocked at a density of ~ 7 g L^−1^ at ~ 10°C and acclimated until 354 DPF in their new aquaria in normoxic conditions before being exposed to either 97.8 ± 2.2% DO at 10.3 ± 0.3°C (normoxia) or 51.2 ± 3.4% DO at 10.3 ± 0.3°C (hypoxia). Water was recirculated to each six-tank system from separate 200-L sumps at 3 L min^−1^ for each tank, and each 200-L sump was recirculated at 3–5 L min^−1^ from a common semi-closed recirculating filtration system. DO in the normoxia system was maintained with vigorous aeration, and DO in the hypoxia treatment system was maintained by nitrogen injection into the 200-L sump controlled by an OxyGuard Pacific oxygen monitoring system (OxyGuard, Denmark). Water temperature was held at ~10°C by a heat exchanger controlled by a central building management system. Water quality was monitored on at least 3 days per week and maintained at 0–0.18 mg L^−1^ NH_3_-N (ammonia), 0.07–0.18 mg L^−1^ NH_2_-N (nitrite), 7.15–7.75 pH and 35–60 mg L^−1^ CaCO_3_ (alkalinity). Fish were reared under the acclimation conditions for ~33 days and fed to satiation once per day with a commercial pellet feed (as above).

### Haematology during experimental acclimations

Haematocrit and Hb] were measured in 53–58 hypoxia-acclimated fish at 0 days (pre-treatment) and 1, 7 and 14 days of hypoxia exposure and in 54–60 normoxia-acclimated fish at 0, 7 and 14 days following establishment of experimental acclimation treatments. Approximately equal numbers of fish were haphazardly selected from each replicate tank. Each fish was euthanized by bathing it in an overdose of well-aerated anaesthetic solution (Aqui-S, Lower Hutt, New Zealand) for 10–20 min prior to blood sampling. Blood was obtained by transecting the caudal peduncle using a sterilized scalpel blade, and the initial pool of blood was removed with paper towel to avoid potential contamination caused by removal of the tail. Subsequently, blood for analysis was sampled directly from the blood pooling on the transected caudal peduncle into a 0.8-mm-diameter heparinized microcapillary tube (Drummond Scientific Co., USA) for haematocrit measurement and a HemoCue Hb201+ microcuvette (HemoCue, Ängelholm, Sweden) for haemoglobin measurement. Haematocrit was calculated as the percentage of packed red cell volume in whole blood following centrifugation at ~ 4000 RCF for 4 min using a microhaematocrit centrifuge (SpinCrit, USA). Blood [Hb] determined by the HemoCue (following 7 min of incubation in the microcuvette; see Clark *et al.* (2008)) was adjusted using a calibration equation for Atlantic salmon blood (Andrewartha *et al.*, 2016). Mean corpuscular haemoglobin concentration (MCHC) was calculated from [Hb] and haematocrit using Equation 1.

(1) \begin{equation*} \mathrm{MCHC}=\frac{\left[\mathrm{Hb}\right]}{\left(\mathrm{Haematocrit}\div 100\right)} \end{equation*}

### Respirometry setup

Aerobic metabolic rate (*Ṁ*O_2_: oxygen uptake rate) of individual fish (10.18 ± 0.24 g) was measured in 552-mL (total volume) intermittent-flow respirometers with constant mixing. Each respirometer consisted of a plastic chamber with an o-ring sealed lid. Oxygen concentration was measured at 5-s intervals using an optical oxygen sensor connected to a four channel FireStingO2 optical oxygen meter (PyroScience, Germany). The oxygen sensor for each respirometer was mounted within the outflow pipe of a small submersible pump inside the respirometer to shield the sensor from contact with the fish. The pump ensured that the respirometer water remained constantly mixed and homogenous. Fifteen respirometry chambers were submerged in a single water bath that was housed in a temperature-controlled room and supplied with freshwater from a common sump. The sump temperature was maintained at ~10°C with a submersible titanium heater (Aqua Logic, CA, USA) and DO was maintained at either 100% by vigorous aeration, or at 50% by nitrogen injection controlled by an OxyGuard Atlantic oxygen monitoring system (OxyGuard International, Denmark). Water for all respirometers was replaced every 10 min via a flush pump with a single timer-controlled solenoid valve that controlled water flow.

### Respirometry protocol

Respirometry measurements commenced 33 days after establishing the experimental acclimation treatments (388 DPF) and were completed by 44 days post-exposure (Fig. 1). Fish were fasted for at least 18 h prior to respirometry. Fish were measured from a total of eight incubation/acclimation treatment group combinations (Table 1). These groups will be referred to as ‘incubation treatment’ + ‘acclimation treatment’ + ‘DO at respirometry measurements’. Note that the normoxia incubation + normoxia acclimation + 50% DO measurement and the normoxia incubation + hypoxia acclimation + 100% DO measurement groups were included to test the acute responses of fish to a change in DO immediately upon removal from their acclimation conditions. Each respirometry trial was conducted over 1 day at either 50% or 100% DO, so that all respirometers in a trial could be maintained under the same DO conditions.

**Table 1:** Incubation treatment, acclimation treatment and respirometry measurement DO group combinations and sample sizes for Atlantic salmon (*Salmo salar*) respirometry measurements

Incubation treatment	Acclimation treatment	Respirometry measurement DO (%)	Sample size
Normoxia	Normoxia	100	19
Normoxia	Normoxia	50	14
Normoxia	Hypoxia	100	13
Normoxia	Hypoxia	50	19
Constant hypoxia	Normoxia	100	20
Constant hypoxia	Hypoxia	50	16
Cyclical hypoxia	Normoxia	100	19
Cyclical hypoxia	Hypoxia	50	19

Fish were haphazardly selected from each incubation + acclimation treatment group, ensuring that approximately equal numbers were selected from each replicate acclimation treatment tank (Table 1). Individual fish were transferred to respirometers (at respective acclimation conditions) and allowed at least 60 min to settle prior to undergoing an exhaustive exercise protocol to induce *Ṁ*O_2max_ as described previously (Clark *et al.*, 2013; Norin and Clark, 2016). Briefly, fish were individually transferred from the respirometers to a 44-L cylindrical exercise tank (69 cm diameter × 12 cm water depth) that was constantly replenished with water from the water bath containing the respirometers to ensure constant DO and temperature. Fish were chased by hand for 60 s using tail tapping to encourage burst swimming. All fish stopped burst swimming within 60s, indicating they had reached exhaustion, but were forced to continue exercising maximally for the entire time period. Following the exercise protocol, the fish were immediately (within 15 s) sealed in their respirometers and DO was measured every 5 s until it dropped by a maximum of 15% DO, at which point the respirometer was flushed. After the final *Ṁ*O_2max_ measurement for each run was complete, all respirometers were set to a 10:10 min flush:seal cycle for at least 13 h (including overnight) to determine the post-absorptive resting metabolic rate (*Ṁ*O_2min_).

### O_2crit_, LOE and post-respirometry haematology

At completion of the *Ṁ*O_2min_ measurements, the respirometers were sealed and oxygen was allowed to decline as it was consumed by the fish. Each fish was constantly monitored, and at LOE (ventral surface of fish visible for at least 5 s), the DO was recorded and the chamber was set to continuously flush for at least 10 min while the fish recovered equilibrium. The time elapsed between sealing the chamber and LOE varied between 51 and 416 min. The critical oxygen tension (O_2crit_; the DO below which *Ṁ*O_2min_ cannot be maintained) always occurred at a higher DO than LOE. Following the recovery of equilibrium, each fish was removed from the respirometer, a blood sample was taken for haematocrit and [Hb] measurements (as described above) and the chambers were sealed for at least 40 min to measure background respiration.

Additionally, O_2crit_ and DO at LOE were measured in a subset of fish using a modified approach to reduce CO_2_ and metabolite build-up in the respirometers and investigate any influences on O_2crit_ and DO at LOE. In the modified approach, DO was gradually decreased via nitrogen injection over a 90-min period while simultaneously measuring *Ṁ*O_2_ during a 10:10 min flush:seal cycle. The respirometers were sealed at 100, 90, 80, 70 and 60% DO for *Ṁ*O_2_ measurements, and DO in the respirometry sump was decreased to the next measurement level during each 10-min sealed cycle by injecting nitrogen gas. Once DO decreased to 50% DO, the chambers were sealed and DO allowed to decline via fish respiration until LOE; subsequently, the fish were sampled as described above.

### Data analysis and statistics

Oxygen uptake rate (*Ṁ*O_2_, mg O_2_ min^−1^) was calculated from the rate of declining DO using Equation 2.

(2) \begin{align*} {\dot{M\mathrm{O}}}_2\left(\mathrm{mg}\ {\mathrm{O}}_2{\min}^{-1}\right)=&\ \frac{\Delta \ \mathrm{DO}}{\Delta \ t}\times \left({P}_{\mathrm{B}}-\left({P}_{\mathrm{S}}\times \mathrm{RH}\right)\right)\nonumber\\ &\times{\beta}_{O_2}\times \mathrm{vol}\times 0.2094 \end{align*}

where DO is the fractional DO saturation, *t* is time in minutes, *P*_B_ is the barometric pressure (kPa), *P*_S_ is the calculated saturation vapour pressure of water (kpa; Antoine equation), RH is the fractional relative humidity, βO_2_ is the oxygen capacitance of water (~0.5375 mg L^−1^ kPa^−1^; see Dejours (1981)) and vol is the volume of the respirometry chamber minus fish volume in L (assuming 1 kg wet fish mass = 1 L volume).

The mean background respiration of all chambers for each run was subtracted from all fish *Ṁ*O_2_ measurements for that run. Background respiration did not exceed 15% of fish *Ṁ*O_2_ for all runs except 4 and 5, where background respiration was ~37% and 32% of fish *Ṁ*O_2_, respectively. For the resting period of the respirometry protocol, a slope between 360 and 510 s long was used for each sealed event to calculate *Ṁ*O_2_. The minimum *Ṁ*O_2_ (*Ṁ*O_2min_) for each individual was calculated as the mean of the lowest four *Ṁ*O_2_ values (from a total of 40–47 measurements per fish), after excluding exceedingly high or low values that were outside ±2 SD of the mean of the lowest four values (Clark *et al.*, 2013). Post-exercise *Ṁ*O_2_ was calculated from the steepest 60-s declining DO slope following exhaustive exercise, and *Ṁ*O_2max_ was calculated from the steepest slope obtained at any point throughout the ~ 15-h respirometry protocol. Aerobic scope was calculated for each individual as *Ṁ*O_2max_ − *Ṁ*O_2min_.

For the duration of the LOE trial, *Ṁ*O_2_ was calculated for each consecutive 360-s interval. O_2crit_ was calculated using the calcO_2_Crit function within the fishMO2 R package with our measurements of *Ṁ*O_2min_ and *Ṁ*O_2_ in a declining oxygen environment (Chabot, 2016; Claireaux and Chabot, 2016). In brief, the function calculates O_2crit_ by fitting a linear regression to the *Ṁ*O_2_ values measured below the lowest DO where the *Ṁ*O_2_ is greater than the fifth percentile of all *Ṁ*O_2min_ measurements calculated previously (pivotDO). O_2crit_ is then determined as the DO where the linear regression intercepts *Ṁ*O_2min_. To suit our experiment, pivotDO was calculated from *Ṁ*O_2_ values measured at >40% DO for fish that commenced at 50% DO and at >80% DO for fish that commenced at 100% DO. Our calculations of O_2crit_ used *Ṁ*O_2min_ determined via the abovementioned approaches rather than using the calcSMR function included within the fishMO2 package.

In addition, acute hypoxia tolerance was defined as the cumulative oxygen deficiency between O_2crit_ and LOE. Oxygen deficiency was calculated from a function between time and DO below O_2crit_ before LOE (DO_def_; Equation 3 (Claireaux and Chabot, 2016).

(3) \begin{equation*} {\mathrm{DO}}_{\mathrm{def}}=\sum_{t=0}^{t=\mathrm{end}}\left(\frac{{\mathrm{O}}_{2\mathrm{crit}}-{\mathrm{DO}}_t}{60}\right) \end{equation*}

where *t* = 0 is time when DO drops below O_2crit_, *t* = end is the time when the fish loses equilibrium and time increment = 1 min. For example, 1 h spent at 1% DO below O_2crit_ is equivalent to a DO_def_ of 1.

All statistical analyses were performed using R and the packages lme4, lsmeans and car (Fox and Weisberg, 2011; Bates *et al.*, 2015; Lenth, 2016; R Core Team, 2016). Differences in *Ṁ*O_2min_, *Ṁ*O_2max_, aerobic scope, O_2crit_, DO at LOE and DO_def_ between incubation treatment, acclimation treatment and measurement treatment groups were tested using ANCOVA (Type III SS) with fish mass as a covariate or using ANOVA (Type III SS) when there was not a significant relationship with fish mass. Where significant covariate interactions were detected, the independent variable and covariate (mass) were log-transformed to satisfy the statistical assumptions of ANCOVA and allow robust group comparisons. Differences in fish mass between incubation treatment groups, acclimation treatment groups and measurement treatment groups were tested using ANOVA (Type III SS). The effect of incubation and acclimation DO on [Hb], haematocrit and MCHC was tested using a linear mixed effects model with tank as the random effect and *P* values computed using Kenward–Roger approximations with Type III SS. Pairwise comparisons were conducted using least square means and either the Tukey method (ANCOVA, ANOVA) or Kenward–Roger approximations (linear mixed effects model). Means are reported as mean ± SEM unless otherwise stated and comparisons of means from ANCOVA analyses calculated using least square means.

## Results

### Changes in haematology during acclimation

**Figure 2:**
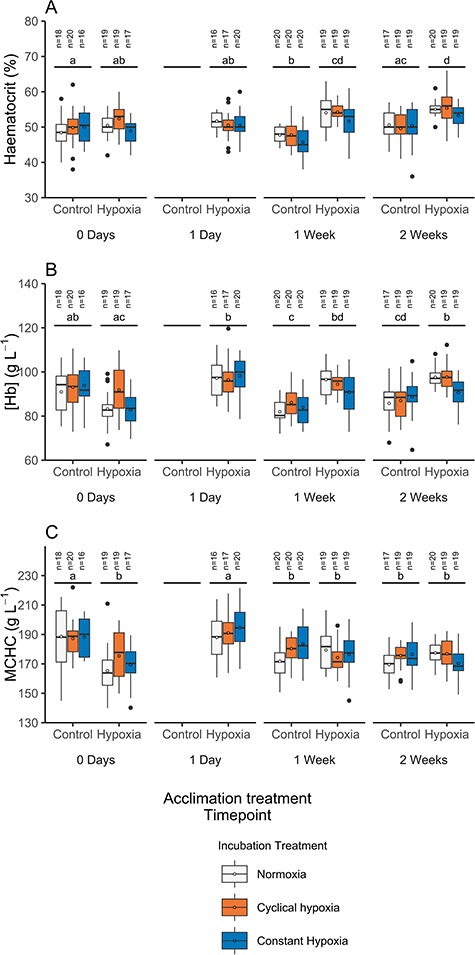
Boxplots of haematocrit (A), [Hb] (B) or MCHC (C) in normoxia and hypoxia-acclimated Atlantic salmon (*Salmo salar*) incubated in normoxia, constant hypoxia (~63% DO) or cyclical hypoxia (100–25% DO daily). Fish acclimated to normoxia were not measured at Day 1 of the acclimation period. The solid central line is the median, the box denotes 25th and 75th percentile, whiskers extend to the highest or lowest value within 1.5*inter-quartile range and filled circles are outliers beyond that range. Mean values are represented by open circles. Different lowercase letters represent significant differences between time-points across hypoxia and normoxia acclimation treatments (averaged across incubation treatment; Tukey, *P* < 0.05).

Haemoglobin concentration ([Hb]), haematocrit and MCHC were not affected by the previous incubation treatment (i.e. constant or cyclical hypoxia or normoxia) at any time-point across both acclimation treatments (normoxia and hypoxia; Fig. 2, Table S3). Haemoglobin concentration, haematocrit and MCHC were each affected by acclimation treatment dependent on measurement time-point (acclimation × time-point interaction; Table S3), and as such, post hoc tests were conducted to provide pairwise comparisons. Prior to commencing the hypoxia acclimation (0 days), [Hb] (89.3 ± 1.0 g L^−1^), haematocrit (50.0 ± 0.4%) and body mass (5.84 ± 0.23 g) were similar between the groups destined for normoxia and hypoxia acclimation (*P* = 0.1403, *P* = 0.9570 and *P* = 0.1979, respectively, Fig. 2A and B). At Day 0, MCHC was ~ 10% higher in the group destined for normoxia acclimation vs. hypoxia acclimation (188.0 ± 2.1 vs. 170.0 ± 2.2 g L^−1^, respectively, P = 0.0001, Fig. 2C).

Mean haemoglobin concentration of hypoxia-acclimated fish increased ~ 13% from 0 days to 1 day post-exposure (*P* < 0.0001) and was elevated above normoxia-acclimated fish at 1 and 2 weeks (both *P* < 0.0001, Fig. 2B). Haematocrit was similar within the hypoxia acclimation group at 0 days and 1 day post-hypoxia exposure (*P* > 0.05); however, it was ~6–8% higher at 1 and 2 weeks relative to 0 days (*P* = 0.0075 and *P* < 0.0001, respectively, Fig. 2A) and ~ 9–13% higher relative to normoxia-acclimated fish at 1 and 2 weeks (*P* = 0.0012 and *P* = 0.0208, respectively, Fig. 2A). Consequently, MCHC initially increased by ~13% after 1 day in hypoxia (*P* < 0.0001) but at 1 and 2 weeks returned to levels similar to pre-hypoxia exposure (0 days) and normoxia-acclimated fish (*P* > 0.05, Fig. 2C). Notably, fish acclimated to normoxia were larger at 2 weeks of acclimation than those acclimated to hypoxia (9.38 ± 0.55 g vs. 7.53 ± 0.40 g, respectively, *P* = 0.0021).

### Metabolism, hypoxia tolerance and haematology following acclimation

The mass of fish used in metabolic experiments (after ~5 weeks of acclimation) was similar between incubation, acclimation and measurement treatment groups (13.41 ± 0.88 g). Incubation treatment had no effect on *Ṁ*O_2min_, *Ṁ*O_2max_, aerobic scope, O_2crit_, DO at LOE or DO_def_ of fish when acclimated and measured in either hypoxia or normoxia (Table S1, Figs S2 and S3). As such, the incubation treatment groups were combined for subsequent analyses to test for effects of acclimation and measurement treatment conditions.

**Figure 3:**
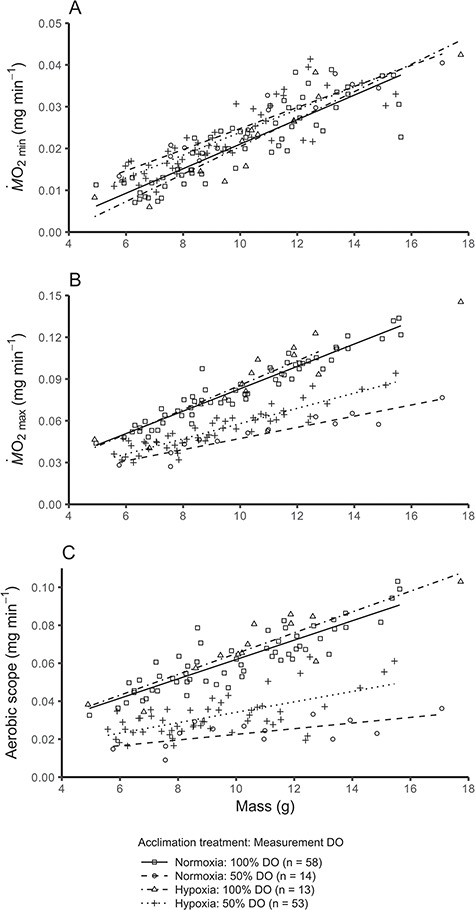
Relationships between mass and *Ṁ*O_2min_ (A), *Ṁ*O_2max_ (B) or aerobic scope (C) in Atlantic salmon (*Salmo salar*) acclimated to either hypoxia or normoxia and then measured in 50 or 100% DO. Because there was no difference between incubation treatment groups, they were pooled (all incubation treatment groups are displayed in Fig. S2). Data points represent individuals, and lines are linear relationships for each acclimation + measurement group combination. In (A), 50% DO >100% DO (acclimation treatments pooled, *P* < 0.05), in (B) and (C) normoxia: 100% DO = hypoxia 100% DO > hypoxia: 50% DO > hypoxia 50% DO (pairwise comparisons, *P* < 0.05)

Minimum *Ṁ*O_2_ was ~ 16% higher (comparison of least-square means) in fish measured in 50% DO compared with fish measured in 100% DO, while acclimation treatment had no effect (P = 0.0003 and 0.6340, respectively; Fig. 3A and Table S2A). Exposure to 50% DO during respirometry caused general reductions in *Ṁ*O_2max_ and aerobic scope compared with measurements conducted at 100% DO. However, when measured at 50% DO, hypoxia-acclimated fish had ~ 23% higher *Ṁ*O_2max_ and ~ 52% higher aerobic scope than normoxia-acclimated fish (both *P* < 0.0001; Fig. 3B and C). Interestingly, *Ṁ*O_2max_ and aerobic scope were similar between hypoxia-acclimated and normoxia-acclimated fish when measured at 100% DO (*P* = 0.9074 and *P* = 0.8684 respectively; Fig. 3B and C).

**Figure 4:**
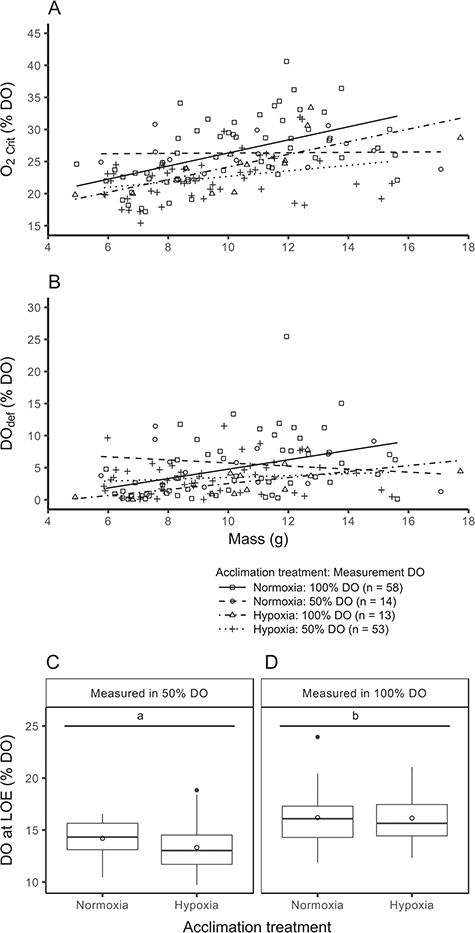
Relationships between mass and O_2crit_ (A) or DO_def_ (B), along with boxplots of DO at LOE (C, D) in Atlantic salmon (*Salmo salar*) acclimated in hypoxia or normoxia and then measured in 50 or 100% DO. Because there was no difference between incubation treatment groups, they were pooled (all incubation treatment groups are displayed in Fig. S3). For scatterplots (A, B), data points represent individuals and lines are linear relationships for each treatment group combination. For boxplots (C, D), the solid central line is the median, the box denotes the 25th and 75th percentiles, whiskers extend to the highest or lowest value within 1.5*inter-quartile range and filled circles are outliers beyond that range. Mean values are represented by open circles. Different lowercase letters indicate significant differences between groups (C, D; Tukey, *P* < 0.05).

**Figure 5:**
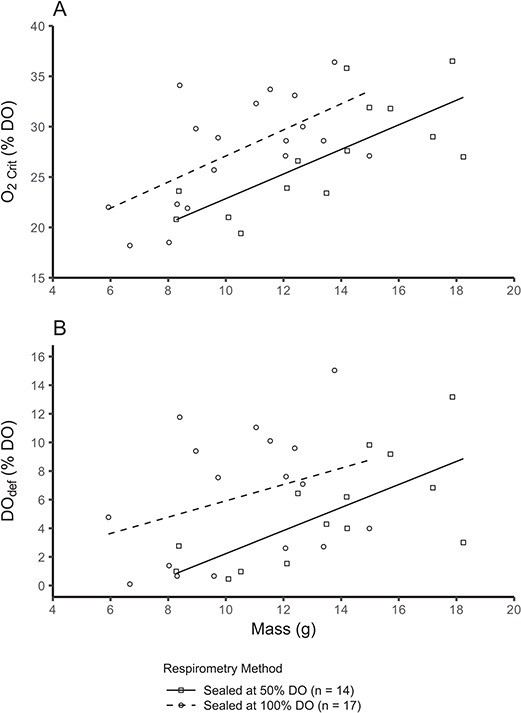
Relationships between mass and O_2crit_ (A) or DO_def_ (B) in Atlantic salmon (*Salmo salar*) during two methods of lowering DO (sealed in respirometers at 100% DO or lowering DO to 50% using nitrogen prior to sealing). Data points represent individual fish, and lines are linear relationships for each treatment group. In (A) and (B), sealed at 100% DO > sealed at 50% DO (pairwise comparisons, *P* < 0.05).

A positive relationship existed between mass and O_2crit_ following log transformation of the data (*P* < 0.0001), which was similar between acclimation treatment groups (*P* = 0.4797, Fig. 4A and Table S2A). However, the relationship between mass and O_2crit_ was different between measurement DO groups (*P* = 0.0072, Fig. 4A and Table S2A), whereby the slope was greater for fish that commenced measurement at 100% DO compared with fish that commenced measurement at 50% DO. Notably, fish that were sealed in respirometers at 100% DO had a higher O_2crit_ than those normoxia-acclimated fish for which DO was lowered to 50% prior to sealing the respirometers (29.3 ± 1.0 vs. 24.9 ± 1.2% DO, lsmeans; *F*_(1,29)_ = 7.10, *P* = 0.0124; Fig. 5A).

The DO at LOE also did not differ between acclimation groups, but it was lower for fish that started at 50% DO compared with those starting at 100% DO (*P* < 0.0001, Fig. 4C and D and Table S2A). Moreover, DO at LOE was similar for normoxia-acclimated fish regardless of whether the respirometers were sealed at 100% DO or reduced to 50% DO via nitrogen injection prior to sealing (*F*_(1,28)_ = 0.017, *P* = 0.8981). Conversely, DO_def_ was lower for fish acclimated to hypoxia compared with normoxia (*P* = 0.0187), but similar between fish measured in 50% DO vs. 100% DO (P = 0.377, Fig. 4B and Table S2A). Normoxia-acclimated fish that were sealed in respirometers at 100% DO had a higher DO_def_ than those for which DO was lowered to 50% prior to sealing the respirometers (7.1 ± 1.0 vs. 3.9 ± 1.1, Fig. 5B, *F*_(1,28)_ = 4.64, *P* = 0.040).

Following respirometry, [Hb] and haematocrit were similar for all acclimation and measurement treatment groups ([Hb]: 96.38 ± 0.79 g L^−1^, haematocrit: 55.16 ± 0.44%, *P* > 0.05, Table S2B). However, an interaction existed for MCHC between acclimation and measurement treatment groups, whereby fish acclimated to hypoxia and measured in 100% DO had a higher MCHC than those acclimated to normoxia and measured in 100% DO (184.0 ± 2.7 vs. 172.1 ± 2.2 g L^−1^, respectively, *P* = 0.0404, Table S2B). When measured in 50% DO, MCHC was similar between fish acclimated to either normoxia or hypoxia (179.3 ± 3.5 vs. 174.1 ± 2.1 g L^−1^, *P* > 0.05).

## Discussion

Despite the potential for incubation conditions to influence the developmental trajectory of fishes, we found no lasting effects of incubation hypoxia on the physiological phenotype (including acclimation capacity) of juvenile Atlantic salmon (Figs. S2 and S3) (Scott and Johnston, 2012; Johnston *et al.*, 2013; Seebacher *et al.*, 2014). In addition, we demonstrated that the severity of reduction in aerobic capacity when measured at 50% DO in the juvenile life stage can be mitigated following ~ 33 days of hypoxia acclimation (Fig. 3C). This compensatory capacity in aerobic performance may be critical to maintaining high performance in the diverse environments that salmonids occupy throughout their lifecycle. Interestingly, the improved aerobic capacity of hypoxia-acclimated fish in 50% DO did not translate to improved performance in 100% DO (Fig. 3C), suggesting that juvenile Atlantic salmon at 100% DO are not oxygen-limited.

### Long-term effects of incubation hypoxia

Most of the relatively few studies that have investigated how the incubation environment impacts long-term acclimation capacity in fish have focused on temperature. Indeed, incubation temperature has been reported to determine the long-term acclimation capacity of U_crit_ in zebrafish as well as *Ṁ*O_2min_ and aerobic scope in mosquitofish (Scott and Johnston, 2012; Seebacher *et al.*, 2014). It is hypothesized that an evolutionary advantage to alter long-term acclimation capacity in response to the incubation environment only exists if the developmental environment provides reliable information about future environments (Beaman *et al.*, 2016). This may help to explain the lack of incubation effects observed in the present study. The DO levels experienced by salmon eggs and alevins in under-gravel redds are not influenced by the same mechanisms as the DO in the freshwater and marine ecosystems inhabited by juveniles and adults (Youngson *et al.*, 2004; Miller *et al.*, 2008; Jenny *et al.*, 2016; Schmidtko *et al.*, 2017). Therefore, the DO concentrations experienced in under-gravel redds are unlikely to provide reliable information about the DO conditions experienced in later life and it would not be advantageous to adjust long-term physiology based on present conditions.

Our results corroborate previous studies reporting no long-term effects of developmental hypoxia on *Ṁ*O_2min_, *Ṁ*O_2max_ and hypoxia tolerance in European seabass and Atlantic salmon or *Ṁ*O_2min_ in zebrafish (Robertson *et al.*, 2014; Vanderplancke *et al.*, 2015; Wood *et al.*, 2017; Zambonino-Infante *et al.*, 2017). However, some evidence exists that incubation hypoxia can cause long-term reductions in aerobic performance of fish. For example, incubation hypoxia reduced the U_crit_ of rainbow trout when measured 50 days post–incubation (Johnston *et al.*, 2013). In the context of the aforementioned studies, our results suggest that the reported impacts of incubation hypoxia on *Ṁ*O_2_ are not sustained during long-term rearing in normoxic conditions.

### Hypoxia acclimation and blood-oxygen carrying capacity

Proportional increases in haematocrit and [Hb] at 1–2 weeks of hypoxia exposure are likely due to an increase in erythropoietin-induced red blood cell formation in an attempt to increase oxygen delivery (Fig. 2) (Lai *et al.*, 2006). However, an increase in [Hb] at 1 day of hypoxia acclimation that was not associated with increased haematocrit was unexpected (Fig. 2). Notably, our blood sampling technique, where fish were sampled after anaesthetic overdose, is likely to have caused splenic contraction and adrenergic-induced red blood cell swelling caused by exercise- and anaesthesia-related stress (Yamamoto, 1987; Pearson and Stevens, 1991; Nikinmaa and Salama, 1998; Hill and Forster, 2004). Therefore, haematocrit values are estimates of maximal rather than baseline or routine levels. While this may preclude comparisons with studies that have used more rapid sampling approaches, it does not impact comparisons across the treatment groups used here (Gallaugher and Farrell, 1998; Clark *et al.*, 2011).

### Hypoxia acclimation and aerobic metabolic capacity

Hypoxia acclimation did not influence *Ṁ*O_2min_ when measured in normoxia or hypoxia, although fish measured in 50% DO exhibited a slightly higher *Ṁ*O_2min_ (Fig. 3A). The latter finding contrasts with our previous study, where *Ṁ*O_2min_ of juvenile Atlantic salmon was not affected when measured in 50% DO and contrasts with a reported decrease in *Ṁ*O_2min_ in rainbow trout exposed to ~50% DO for 24 h (Boutilier *et al.*, 1988; Wood *et al.*, 2017). As such, we view this finding with some caution and suggest that increased activity levels in response to acute hypoxia may be responsible for the apparent elevation in *Ṁ*O_2min_. In any event, the lack of reduction in *Ṁ*O_2min_ at 50% DO is consistent with our finding that O_2crit_ of all individual salmon used here is below 50% DO (Fig. 4A).

Interestingly, we found that ~33–44 days of hypoxia acclimation increased *Ṁ*O_2max_ and aerobic scope of fish when measured in hypoxia (50% DO), but not when measured in normoxia (Fig. 3B and C). As far as we are aware, only one previous study has documented a similar finding, whereby goldfish acclimated to hypoxia for 48 h had a higher *Ṁ*O_2max_ and aerobic scope when measured in hypoxia but not in normoxia (Fu *et al.*, 2011). The increased aerobic scope of hypoxia-acclimated goldfish when measured in hypoxia was associated with an increase in U_crit_, indicating that aerobic activities are likely to improve when oxygen uptake capacity is increased. It is possible that the increased *Ṁ*O_2max_ for hypoxia-acclimated fish when measured in 50% DO (but not in 100% DO; Fig. 3B) may be driven by a leftward shift of the oxygen dissociation curve to enable better oxygen loading at the gills in hypoxia, because hypoxia acclimation can be associated with a decrease in allosteric modulators like blood cell ATP and GTP (Wood and Johansen, 1972; Wood and Johansen, 1973; Tetens and Lykkeboe, 1981; Tetens and Lykkeboe, 1985; Rutjes *et al.*, 2007). Providing some support for that possibility, blood-O_2_ affinity increased while haematocrit and [Hb] were maintained in rainbow trout acclimated to hypoxia for 2 weeks (Bushnell *et al.*, 1984). To further elucidate the differential impacts of hypoxia acclimation on *Ṁ*O_2max_ when measured in hypoxia or normoxia, focus should be placed on the mechanisms that determine oxygen uptake and tissue oxygen demands at an individual level.

Hypoxia acclimation did not improve O_2crit_ or DO at LOE despite the increased *Ṁ*O_2max_ and aerobic scope of hypoxia-acclimated fish in 50% DO, suggesting that hypoxia tolerance is not strongly linked with aerobic capacity (Fig. 4). The lack of improvement in hypoxia tolerance of juvenile Atlantic salmon following hypoxia acclimation contrasts with previous studies on zebrafish (time to death), Atlantic salmon (DO at LOE), barramundi (O_2crit_) and killifish (DO at LOE, O_2crit_), but is similar to studies on Atlantic salmon (O_2crit_) and snapper (*Pagrus auratus*, O_2crit_) (Rees *et al.*, 2001; Cook *et al.*, 2013; Remen *et al.*, 2013; Anttila *et al.*, 2015; Borowiec *et al.*, 2015; Collins *et al.*, 2016). While the factors influencing the acclimation capacity of hypoxia tolerance require further attention, it is clear that any studies investigating links between hypoxia tolerance and aerobic metabolism should additionally examine factors such as anaerobic capacity (Cook *et al.*, 2013).

Notably, O_2crit_ and DO_def_ (but not DO at LOE) were lower when the DO in the respirometers was lowered to 50% using nitrogen injection compared with fish that were subjected to closed respirometry from 100% DO (Fig. 5). Similar findings for shiner perch (*Cymatogaster aggregata*) suggest that a build-up of CO_2_ and metabolites may reduce the hypoxia tolerance of fish (Snyder *et al.*, 2016). However, measuring hypoxia and normoxia acclimation treatment groups in both 50 and 100% DO allows for robust comparisons between acclimation groups.

## Conclusions

Our findings suggest that salmonids exposed to constant hypoxia (~63% DO) or cyclical hypoxia (100–25% DO daily) during early development in under-gravel redds will not experience long-term physiological improvements once they have left the redd. Thus, the impacts of hypoxia in the redd are likely to be more immediate, such as decreased growth and development, and delayed hatching (Hamor and Garside, 1976; Matschak *et al.*, 1997; Miller *et al.*, 2011; Wood *et al.*, 2019). However, hypoxia acclimation reveals marked plasticity in aerobic capacity during the juvenile life stage, which may help to support the energetic requirements of juveniles in environmentally variable freshwater rearing habitats. While increased haematocrit and [Hb] play some role in improving aerobic capacity during hypoxia acclimation, the lack of improvement in aerobic capacity of hypoxia-acclimated fish under normoxic conditions suggests that the performance of juvenile Atlantic salmon at 100% DO is not oxygen-limited. Our findings suggest that there may be little evolutionary advantage in Atlantic salmon modifying their hypoxia acclimation capacity in response to their incubation environment, at least under the levels of constant (~63% DO) and cyclical (100–25% DO daily) incubation hypoxia used here. Instead, it appears that salmonids possess sufficient physiological plasticity to acclimate to the unpredictable oxygen levels encountered throughout their complex lifecycle. However, the Atlantic salmon used in the present study were sourced from a selectively bred domesticated population of Atlantic salmon used for aquaculture in Tasmania, Australia. Physiological traits and plasticity of domesticated Atlantic salmon can differ to wild populations and can also differ between wild populations from different locations (Jensen *et al.*, 2008; Hutchings, 2011; Glover *et al.*, 2017; Cook *et al.*, 2018). We are not aware of any studies that investigate the differences in hypoxia acclimation capacity or physiology between cultured Tasmanian Atlantic salmon and any wild populations. Care should be taken to consider the attributes of local salmon populations and local conditions if applying the results of the present study in conservation and management decisions. Whether or not physiological plasticity in all Atlantic salmon populations can accommodate potentially more severe localized conditions and the increasingly common hypoxia documented in aquatic environments remains a question for conservation physiologists.

## Supplementary Material

Hypoxia_during_incubation_cons_phys_final_revised_coz088

## References

[ref1] Andrewartha SJ, Munns SL, Edwards A (2016) Calibration of the HemoCue point-of-care analyser for determining haemoglobin concentration in a lizard and a fish. Conserv Physiol 4: cow006. 27293758 10.1093/conphys/cow006 PMC4804733

[ref2] Anttila K, Lewis M, Prokkola JM, Kanerva M, Seppanen E, Kolari I, Nikinmaa M (2015) Warm acclimation and oxygen depletion induce species-specific responses in salmonids. J Exp Biol 218: 1471–1477. 25827840 10.1242/jeb.119115

[ref3] Bates D, Maechler M, Bolker B, Walker S (2015) Fitting linear mixed-effects models using lme4. J Stat Softw 67: 1–48.

[ref4] Bateson P, Gluckman P, Hanson M (2014) The biology of developmental plasticity and the predictive adaptive response hypothesis. J Physiol 592: 2357–2368. 24882817 10.1113/jphysiol.2014.271460 PMC4048093

[ref5] Beaman JE, White CR, Seebacher F (2016) Evolution of plasticity: mechanistic link between development and reversible acclimation. Trends Ecol Evol 31: 237–249. 26846962 10.1016/j.tree.2016.01.004

[ref6] Beldade P, Mateus ARA, Keller RA (2011) Evolution and molecular mechanisms of adaptive developmental plasticity. Mol Ecol 20: 1347–1363. 21342300 10.1111/j.1365-294X.2011.05016.x

[ref7] Borowiec BG, Darcy KL, Gillette DM, Scott GR (2015) Distinct physiological strategies are used to cope with constant hypoxia and intermittent hypoxia in killifish (*Fundulus heteroclitus*). J Exp Biol 218: 1198–1211. 25722002 10.1242/jeb.114579

[ref8] Boutilier RG, Dobson G, Hoeger U, Randall DJ (1988) Acute exposure to graded levels of hypoxia in rainbow trout (*Salmo gairdneri*): metabolic and respiratory adaptations. Resp Physiol 71: 69–82. 10.1016/0034-5687(88)90116-8 3340814

[ref9] Burggren WW, Reyna KS (2011) Developmental trajectories, critical windows and phenotypic alteration during cardio-respiratory development. Respir Physiol Neurobiol 178: 13–21. 21596160 10.1016/j.resp.2011.05.001

[ref10] Bushnell PG, Steffensen JF, Johansen K (1984) Oxygen-consumption and swimming performance in hypoxia-acclimated rainbow trout *Salmo gairdneri*. J Exp Biol 113: 225–235.

[ref11] Chabot D (2016). fishMO2: Calculate and plot the standard metabolic rate (SMR), the critical oxygen level (O2crit) and the specific dynamic action (SDA) and related variables in fishes and crustaceans, Ed 0.37

[ref12] Ciuhandu CS, Wright PA, Goldberg JI, Stevens ED (2007) Parameters influencing the dissolved oxygen in the boundary layer of rainbow trout (*Oncorhynchus mykiss*) embryos and larvae. J Exp Biol 210: 1435–1445. 17401126 10.1242/jeb.02754

[ref13] Claireaux G, Chabot D (2016) Responses by fishes to environmental hypoxia: integration through Fry's concept of aerobic metabolic scope. J Fish Biol 88: 232–251. 26768976 10.1111/jfb.12833

[ref14] Clark TD, Donaldson MR, Drenner SM, Hinch SG, Patterson DA, Hills J, Ives V, Carter JJ, Cooke SJ, Farrell AP (2011) The efficacy of field techniques for obtaining and storing blood samples from fishes. J Fish Biol 79: 1322–1333. 22026608 10.1111/j.1095-8649.2011.03118.x

[ref15] Clark TD, Eliason EJ, Sandblom E, Hinch SG, Farrell AP (2008) Calibration of a hand-held haemoglobin analyser for use on fish blood. J Fish Biol 73: 2587–2595.

[ref16] Clark TD, Sandblom E, Jutfelt F (2013) Aerobic scope measurements of fishes in an era of climate change: respirometry, relevance and recommendations. J Exp Biol 216: 2771–2782. 23842625 10.1242/jeb.084251

[ref17] Collins GM, Clark TD, Carton AG (2016) Physiological plasticity v. inter-population variability: understanding drivers of hypoxia tolerance in a tropical estuarine fish. Mar Freshw Res 67: 1575–1582.

[ref18] Cook CJ, Burness G, Wilson CC (2018) Metabolic rates of embryos and alevin from a cold-adapted salmonid differ with temperature, population and family of origin: implications for coping with climate change. Conserv Physiol 6. 10.1093/conphys/cox076 PMC5757644 30613399

[ref19] Cook DG, Iftikar FI, Baker DW, Hickey AJR, Herbert NA (2013) Low-O_2_ acclimation shifts the hypoxia avoidance behaviour of snapper (*Pagrus auratus*) with only subtle changes in aerobic and anaerobic function. J Exp Biol 216: 369–378. 23038727 10.1242/jeb.073023

[ref20] Dhiyebi HA, O'Donnell MJ, Wright PA (2013) Water chemistry in the microenvironment of rainbow trout *Oncorhynchus mykiss* embryos is affected by development, the egg capsule and crowding. J Fish Biol 82: 444–457. 23398061 10.1111/j.1095-8649.2012.03491.x

[ref21] Diaz RJ, Breitburg DL (2009) The hypoxic environment In Richards JG, Farrell AP, Colin JB, eds, Fish Physiology Vol. 27: Academic Press, pp. 1–23.

[ref22] Diaz RJ, Rosenberg R (2008) Spreading dead zones and consequences for marine ecosystems. Science 321: 926–929. 18703733 10.1126/science.1156401

[ref23] Elliott N, Kube P (2009) Development and early results of the Tasmanian Atlantic salmon breeding program. Proc Assoc Advmt Anim Breed Genet 18: 362–365.

[ref24] Fox J, Weisberg S (2011) An R Companion to Applied Regression. Sage, Thousand Oaks, CA.

[ref25] Fry FEJ (1971) The effect of environmental factors on the physiology of fish In Hoar WS, Randall DJ, eds, Fish Physiology Vol. 6: Academic Press, pp. 1–98.

[ref26] Fu S-J, Brauner CJ, Cao Z-D, Richards JG, Peng J-L, Dhillon R, Wang Y-X (2011) The effect of acclimation to hypoxia and sustained exercise on subsequent hypoxia tolerance and swimming performance in goldfish (*Carassius auratus*). J Exp Biol 214: 2080–2088. 21613525 10.1242/jeb.053132

[ref27] Gallaugher P, Farrell AP (1998) Hematocrit and blood oxygen-carrying capacity In Perry SF, Tufts BL, eds, Fish Physiology Vol. 17: Academic Press, pp. 185–227.

[ref28] Gilbert SF (2012) Ecological developmental biology: environmental signals for normal animal development. Evol Dev 14: 20–28. 23016971 10.1111/j.1525-142X.2011.00519.x

[ref29] Glover KA, Solberg MF, McGinnity P, Hindar K, Verspoor E, Coulson MW, Hansen MM, Araki H, Skaala Ø, Svåsand T (2017) Half a century of genetic interaction between farmed and wild Atlantic salmon: status of knowledge and unanswered questions. Fish and Fisheries 18: 890–927.

[ref30] Greig SM, Sear DA, Carling PA (2007) A review of factors influencing the availability of dissolved oxygen to incubating salmonid embryos. Hydrol Process 21: 323–334.

[ref31] Hamor T, Garside ET (1976) Developmental rates of embryos of Atlantic salmon, *Salmo salar* L., in response to various levels of temperature, dissolved oxygen, and water exchange. Can J Zool 54: 1912–1917. 991016 10.1139/z76-221

[ref32] Hill JV, Forster ME (2004) Cardiovascular responses of Chinook salmon (*Oncorhynchus tshawytscha*) during rapid anaesthetic induction and recovery. Comp Biochem Physiol Part C Toxicol Pharmcol 137: 167–177. 10.1016/j.cca.2004.01.002 15050928

[ref33] Hutchings JA (2011) Old wine in new bottles: reaction norms in salmonid fishes. Heredity 106: 421–437. 21224878 10.1038/hdy.2010.166 PMC3131971

[ref34] Ingendahl D (2001) Dissolved oxygen concentration and emergence of sea trout fry from natural redds in tributaries of the River Rhine. J Fish Biol 58: 325–341.

[ref35] Jenny J-P, Francus P, Normandeau A, Lapointe F, Perga M-E, Ojala A, Schimmelmann A, Zolitschka B (2016) Global spread of hypoxia in freshwater ecosystems during the last three centuries is caused by rising local human pressure. Glob Chang Biol 22: 1481–1489. 26666217 10.1111/gcb.13193

[ref36] Jensen LF, Hansen MM, Pertoldi C, Holdensgaard G, Mensberg K-LD, Loeschcke V (2008) Local adaptation in brown trout early life-history traits: implications for climate change adaptability. Proc R Soc B Biol Sci 275: 2859–2868. 10.1098/rspb.2008.0870 PMC2605839 18755673

[ref37] Johnston EF, Alderman SL, Gillis TE (2013) Chronic hypoxia exposure of trout embryos alters swimming performance and cardiac gene expression in larvae. Physiol Biochem Zool 86: 567–575. 23995487 10.1086/672012

[ref38] Keeling RE, Kortzinger A, Gruber N (2010) Ocean deoxygenation in a warming world. Annu Rev Mar Sci 2: 199–229. 10.1146/annurev.marine.010908.163855 21141663

[ref39] Lai JCC, Kakuta I, Mok HOL, Rummer JL, Randall D (2006) Effects of moderate and substantial hypoxia on erythropoietin levels in rainbow trout kidney and spleen. J Exp Biol 209: 2734–2738. 16809464 10.1242/jeb.02279

[ref40] Lenth RV (2016) Least-squares means: the R package lsmeans. J Stat Softw 69: 1–33.

[ref41] Matschak TW, Stickland NC, Mason PS, Crook AR (1997) Oxygen availability and temperature affect embryonic muscle development in Atlantic salmon (*Salmo salar* L.). Differentiation 61: 229–235.

[ref42] Miller SC, Gillis TE, Wright PA (2011) The ontogeny of regulatory control of the rainbow trout (*Oncorhynchus mykiss*) heart and how this is influenced by chronic hypoxia exposure. J Exp Biol 214: 2065–2072. 21613523 10.1242/jeb.054825

[ref43] Miller SC, Reeb SE, Wright PA, Gillis TE (2008) Oxygen concentration in the water boundary layer next to rainbow trout (*Oncorhynchus mykiss*) embryos is influenced by hypoxia exposure time, metabolic rate, and water flow. Can J Fish Aquat Sci 65: 2170–2177.

[ref44] Motyka R, Norin T, Petersen LH, Huggett DB, Gamperl AK (2017) Long-term hypoxia exposure alters the cardiorespiratory physiology of steelhead trout (*Oncorhynchus mykiss*), but does not affect their upper thermal tolerance. J Therm Biol 68: 149–161. 28797475 10.1016/j.jtherbio.2016.03.007

[ref45] Nikinmaa M, Salama A (1998) Oxygen transport in fish In Perry SF, Tufts B, eds, Fish Physiology Vol. 17: Academic Press, pp. 141–184

[ref46] Norin T, Clark TD (2016) Measurement and relevance of maximum metabolic rate in fishes. J Fish Biol 88: 122–151. 26586591 10.1111/jfb.12796

[ref47] Pearson MP, Stevens ED (1991) Size and hematological impact of the splenic erythrocyte reservoir in rainbow trout, *Oncorhynchus mykiss*. Fish Physiol Biochem 9: 39–50. 24214608 10.1007/BF01987610

[ref48] R Core Team (2016) R: a Language and Environment for Statistical Computing, Ed 3.3.1. R Foundation for Statistical Computing, Vienna, Austria

[ref49] Rees BB, Sudradjat FA, Love JW (2001) Acclimation to hypoxia increases survival time of zebrafish, *Danio rerio*, during lethal hypoxia. J Exp Zool 289: 266–272. 11241397 10.1002/1097-010x(20010401/30)289:4<266::aid-jez7>3.0.co;2-5

[ref50] Remen M, Oppedal F, Imsland AK, Olsen RE, Torgersen T (2013) Hypoxia tolerance thresholds for post-smolt Atlantic salmon: dependency of temperature and hypoxia acclimation. Aquaculture 416–417: 41–47.

[ref51] Richards JG (2009) Metabolic and molecular responses of fish to hypoxia In Richards JG, Farrell AP, Colin JB, eds, Fish Physiology Vol. 27: Academic Press, pp. 443–485

[ref52] Robertson CE, Wright PA, Köblitz L, Bernier NJ (2014) Hypoxia-inducible factor- 1 mediates adaptive developmental plasticity of hypoxia tolerance in zebrafish, *Danio rerio*. Proc R Soc B 281. 10.1098/rspb.2014.0637 PMC4046416 24850928

[ref53] Rutjes HA, Nieveen MC, Weber RE, Witte F, Van den Thillart GEEJM (2007) Multiple strategies of Lake Victoria cichlids to cope with lifelong hypoxia include hemoglobin switching. Am J Physiol Regul Integr Comp Physiol 293: R1376–R1383. 17626121 10.1152/ajpregu.00536.2006

[ref54] Schmidtko S, Stramma L, Visbeck M (2017) Decline in global oceanic oxygen content during the past five decades. Nature 542: 335–339. 28202958 10.1038/nature21399

[ref55] Scott GR, Johnston IA (2012) Temperature during embryonic development has persistent effects on thermal acclimation capacity in zebrafish. Proc Natl Acad Sci USA 109: 14247–14252. 22891320 10.1073/pnas.1205012109 PMC3435178

[ref56] Seebacher F, Beaman J, Little AG (2014) Regulation of thermal acclimation varies between generations of the short-lived mosquitofish that developed in different environmental conditions. Funct Ecol 28: 137–148.

[ref57] Shepard MP (1955) Resistance and tolerance of young speckled trout (*Salvelinus fontinalis*) to oxygen lack, with special reference to low oxygen acclimation. J Fish Res Board Can 12: 387–446.

[ref58] Snyder S, Nadler LE, Bayley JS, Svendsen MBS, Johansen JL, Domenici P, Steffensen JF (2016) Effect of closed v. intermittent-flow respirometry on hypoxia tolerance in the shiner perch *Cymatogaster aggregata*. J Fish Biol 88: 252–264. 26768977 10.1111/jfb.12837

[ref59] Tervonen V, Vuolteenaho O, Nikinmaa M (2006) Haemoconcentration via diuresis in short-term hypoxia: a possible role for cardiac natriuretic peptide in rainbow trout. Comp Biochem Physiol Part A Mol Integr Physiol 144: 86–92. 10.1016/j.cbpa.2006.02.014 16603395

[ref60] Tetens V, Lykkeboe G (1981) Blood respiratory properties of rainbow trout, *Salmo gairdneri:* responses to hypoxia acclimation and anoxic incubation of blood in vitro. J Comp Physiol 145: 117–125.

[ref61] Tetens V, Lykkeboe G (1985) Acute exposure of rainbow trout to mild and deep hypoxia: O_2_ affinity and O_2_ capacitance of arterial blood. Resp Physiol 61: 221–235. 10.1016/0034-5687(85)90128-8 4048672

[ref62] Vanderplancke G, Claireaux G, Quazuguel P, Madec L, Ferraresso S, Sévère A, Zambonino-Infante J-L, Mazurais D (2015) Hypoxic episode during the larval period has long-term effects on European sea bass juveniles (*Dicentrarchus labrax*). Mar Biol 162: 367–376.

[ref63] Wood AT, Clark TD, Andrewartha SJ, Elliott NG, Frappell PB (2017) Developmental hypoxia has negligible effects on long-term hypoxia tolerance and aerobic metabolism of Atlantic salmon (*Salmo salar*). Physiol Biochem Zool 90: 494–501. 28459654 10.1086/692250

[ref64] Wood AT, Clark TD, Elliott NG, Frappell PB, Andrewartha SJ (2019) Physiological effects of dissolved oxygen are stage-specific in incubating Atlantic salmon (*Salmo salar*). J Comp Physiol B . 10.1007/s00360-018-1199-5 30603847

[ref65] Wood SC, Johansen K (1972) Adaptation to hypoxia by increased HBO_2_ affinity and decreased red-cell ATP concentration. Nat New Biol 237: 278–279. 4504466 10.1038/newbio237278a0

[ref66] Wood SC, Johansen K (1973) Organic phosphate metabolism in nucleated red cells: influence of hypoxia on eel HbO2 affinity. Neth J Sea Res 7: 328–338.

[ref67] Yamamoto K-I (1987) Contraction of spleen in exercised cyprinid. Comp Biochem Physiol Part A Physiol 87: 1083–1087. 10.1016/0300-9629(87)90043-0 2887374

[ref68] Youngson AF, Malcolm IA, Thorley JL, Bacon PJ, Soulsby C (2004) Long-residence groundwater effects on incubating salmonid eggs: low hyporheic oxygen impairs embryo development. Can J Fish Aquat Sci 61: 2278–2287.

[ref69] Zambonino-Infante JL, Mazurais D, Dubuc A, Queau P, Vanderplancke G, Servili A, Cahu C, Le Bayon N, Huelvan C, Claireaux G (2017) An early life hypoxia event has a long-term impact on protein digestion and growth in juvenile European sea bass. J Exp Biol 220: 1846–1851. 28302867 10.1242/jeb.154922

