# Supplementary material for: Hypoxia during incubation does not affect aerobic performance or haematology of Atlantic salmon (Salmo salar) when re-exposed in later life
Source: Conserv Physiol. 2019 Nov 27;7(1):coz088. doi: 10.1093/conphys/coz088 (PMC6880253; doi:10.1093/conphys/coz088)
Supplement: Hypoxia_during_incubation_cons_phys_final_revised_coz088 [file hypoxia_during_incubation_cons_phys_final_revised_coz088.docx]

## Supplementary Figures and Tables

**
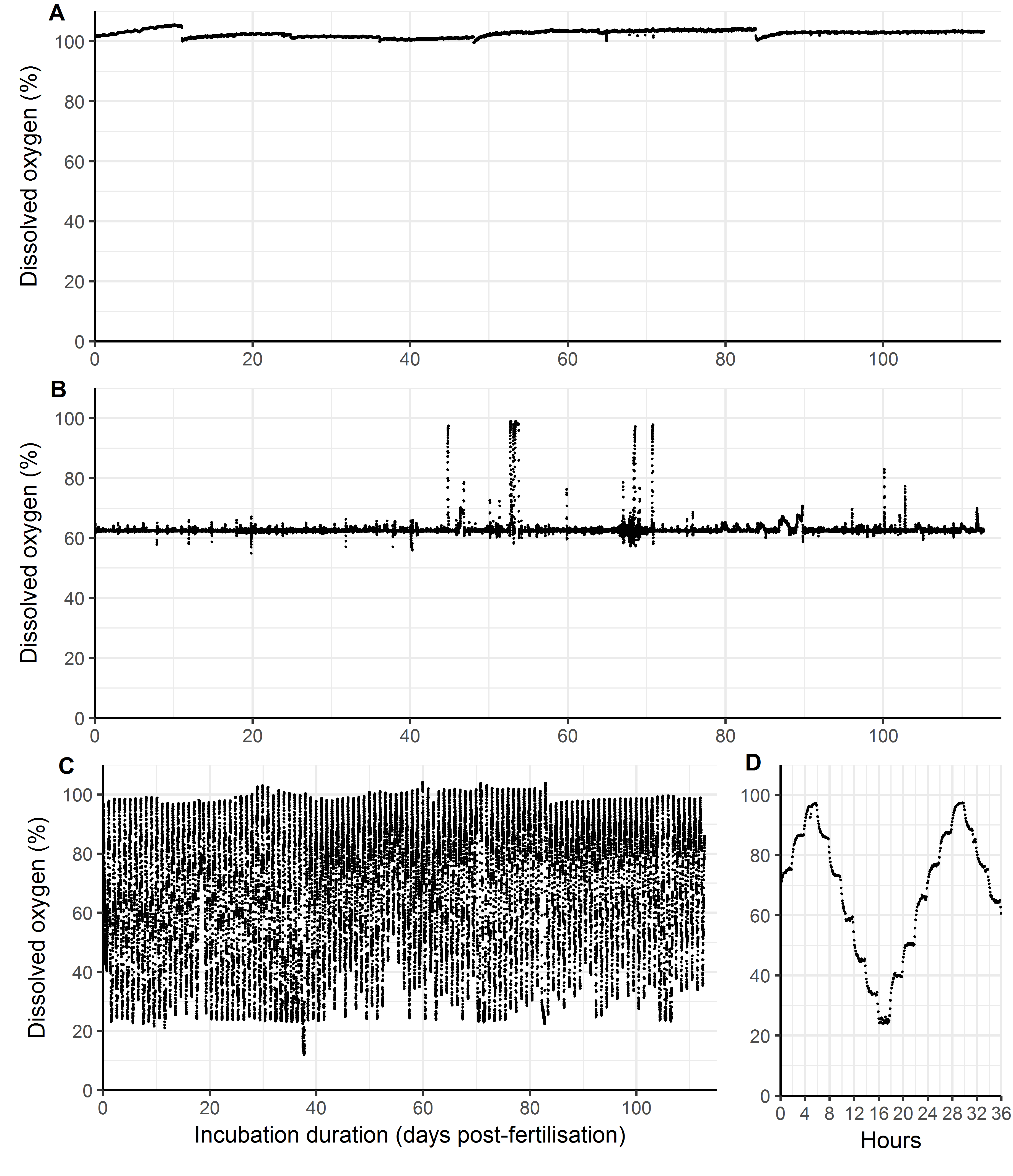
**

**Figure S1:** Dissolved oxygen level (% air saturation) measured within the normoxia (A), constant hypoxia (~63% DO; B) and cyclical hypoxia (100-25% DO daily; C) treatment sumps throughout the incubation treatment period. Panel E shows dissolved oxygen measurements from the cyclical hypoxia treatment sump for a 36 hour period showing a typical hypoxia cycle repeated throughout incubation.


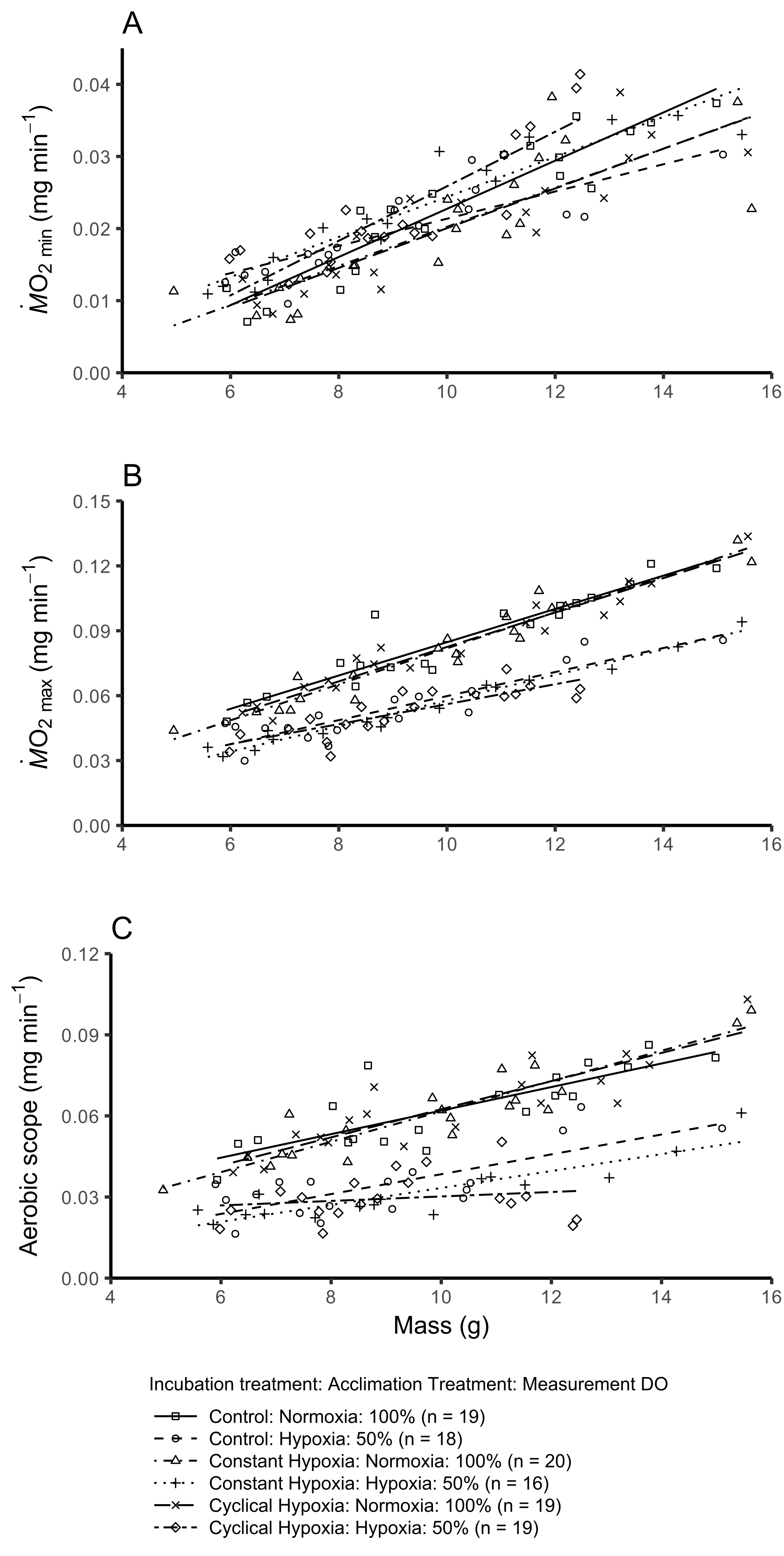


**Figure S2:** The relationship between fish mass and *Ṁ*O_2min_ (A), *Ṁ*O_2max_ (B) and aerobic scope (C). Individuals are represented by data points and lines are relationships between mass and metabolic parameters for each treatment group.


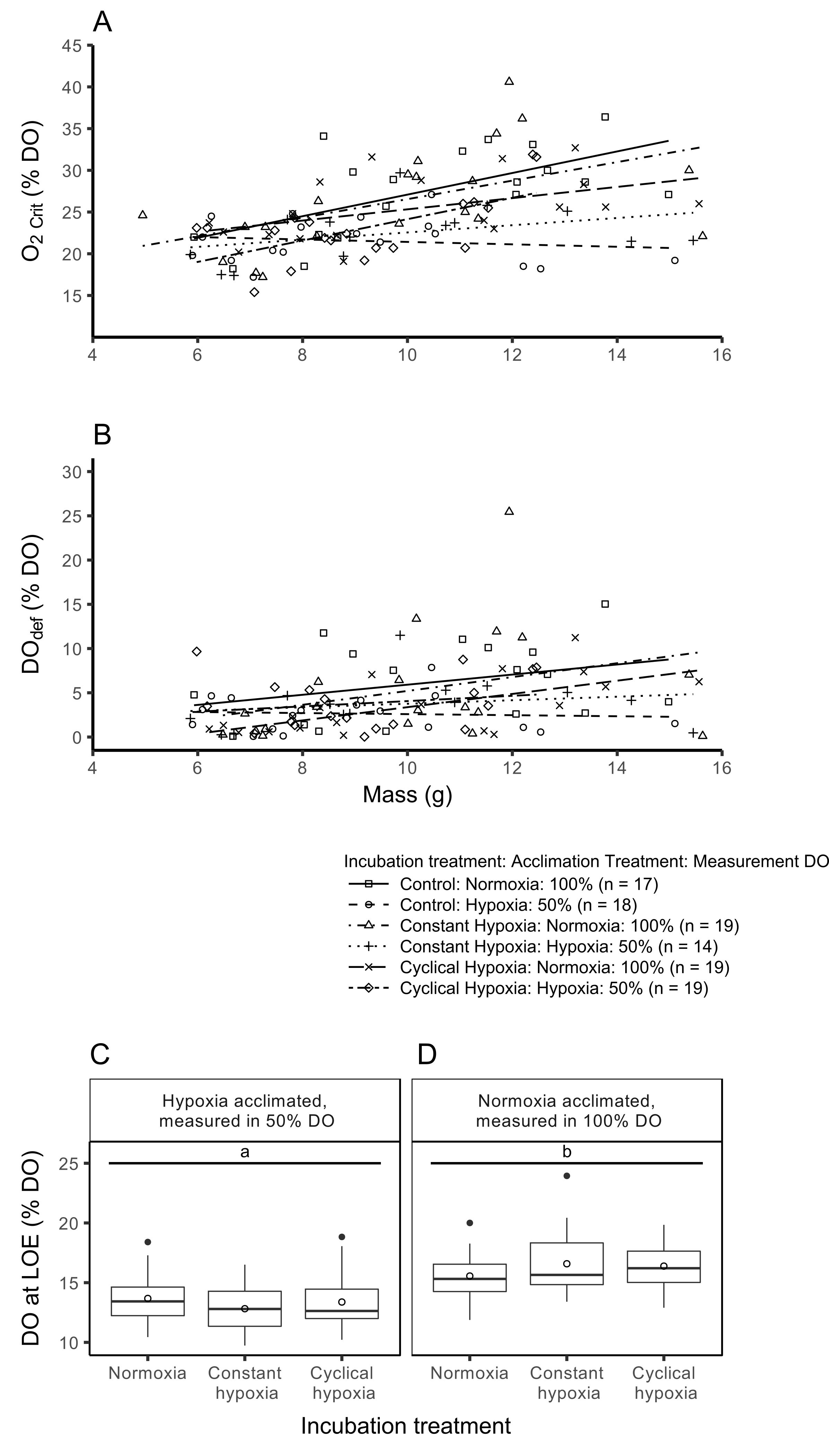


**Figure S3:** The relationship between fish mass and O_2crit_ (A), DO at LOE (B) and DO_def_ (C). Data points represent individual fish and lines are linear relationships between mass and metabolic parameters for each incubation/acclimation treatment group combination. Incubation treatment group had no effect on either O_2crit_, DO at LOE or DO_def_ (P > 0.05).

**Table S1:** Results from ANCOVA (A) and ANOVA (B) testing the effect of incubation treatment and acclimation/measurement treatment on metabolic parameters and haematological parameters following respirometry measurements. Subscript values indicate group and residual degrees of freedom. Highest order effects (P < 0.05) are in bold.

| A | *Ṁ*O_2min_* | | *Ṁ*O_2max_* | | Aerobic Scope* | | O_2_Crit | | DO at LOE^ | | DO_def_ | |
| --- | --- | --- | --- | --- | --- | --- | --- | --- | --- | --- | --- | --- |
|  | *F_104_* | *P* | *F_104_* | *P* | *F_104_* | *P* | *F_101_* | *P* | *F_101_* | *P* | *F_99_* | *P* |
| Mass_1_ | 341.49 | < 0.0001 | 691.54 | < 0.0001 | 123.30 | < 0.0001 | 25.22 | < 0.0001 | - | - | 9.48 | 0.0027 |
| Incubation_2_ | 0.12 | 0.8857 | 1.95 | 0.1478 | 1.86 | 0.1602 | 0.07 | 0.9315 | 0.13 | 0.8770 | 0.45 | 0.6399 |
| Acclimation_1_ | 20.29 | **< 0.0001** | 333.74 | **< 0.0001** | 272.93 | **< 0.0001** | 19.70 | **< 0.0001** | 43.14 | **< 0.0001** | 2.41 | 0.1239 |
| Incubation x Acclimation_2_ | 2.85 | 0.0625 | 0.19 | 0.8257 | 1.98 | 0.1436 | 1.72 | 0.1839 | 1.51 | 0.2257 | 2.11 | 0.1261 |
|  |  | |  | |  | |  | |  | |  | |
| B | Haemoglobin | | Haematocrit | | MCHC | |  | | | | | |
|  | *F_104_* | *P* | *F_88_* | *P* | *F_87_* | *P* |  |  |  |  |  |  |
| Incubation_2_ | 0.44 | 0.6447 | 0.83 | 0.4397 | 0.21 | 0.8087 |  |  |  |  |  |  |
| Acclimation_1_ | 1.35 | 0.2472 | 0.83 | 0.3650 | 0.12 | 0.7302 |  |  |  |  |  |  |
| Incubation x Acclimation_2_ | 2.00 | 0.1405 | 0.25 | 0.7812 | 2.29 | 0.1073 |  |  |  |  |  |  |

*ANCOVA was performed using log transformed independent variable and covariate (mass).

^ANOVA was performed as there was no overall relationship with mass.

**Table S2**: Results of ANCOVA (A) and ANOVA (B) testing for effect of acclimation treatment and measurement oxygen level on metabolic parameters and haematological parameters following respirometry measurements. The highest order effects (P < 0.05) are in bold. Subscript values indicate group and residual degrees of freedom. Dashes indicate that the covariate interactions are not significant and were removed from the model to conduct between groups comparisons.

| A | *Ṁ*O_2min_ | | *Ṁ*O_2max_* | | Aerobic Scope* | | O_2_Crit* | | DO at LOE^ | | DO_def_ | |
| --- | --- | --- | --- | --- | --- | --- | --- | --- | --- | --- | --- | --- |
|  | *F_133_* | *P* | *F_133_* | *P* | *F_133_* | *P* | *F_127_* | *P* | *F_129_* | *P* | *F_127_* | *P* |
| Mass_1_ | 449.13 | < 0.0001 | 830.36 | < 0.0001 | 151.29 | < 0.0001 | 21.72 | < 0.0001 | - | - | 9.14 | 0.0030 |
| Acclimation_1_ | 0.23 | 0.6340 | 24.76 | < 0.0001 | 28.69 | < 0.0001 | 1.32 | 0.2524 | 0.89 | 0.3464 | 5.68 | **0.0187** |
| Measurement_1_ | 13.56 | **0.0003** | 420.30 | < 0.0001 | 381.93 | < 0.0001 | 6.58 | 0.0115 | 24.21 | **< 0.0001** | 0.78 | 0.3776 |
| Mass x Acclimation_1_ | - | - | - | - | - | - | 0.50 | 0.4797 | - | - | - | - |
| Mass x Measurement_1_ | - | **-** | - | - | - | - | 7.46 | **0.0072** | - | - | - | - |
| Acclimation x measurement_1_ | 0.01 | 0.9032 | 15.90 | **0.0001** | 17.87 | **< 0.0001** | 1.02 | 0.3149 | 0.72 | 0.3995 | 0.14 | 0.7047 |
| Mass x acclimation x measurement_1_ | - | - | - | - | - | - | 0.77 | 0.3827 | - | - | - | - |
|  |  | |  | |  | |  | |  | |  | |
| B | Haemoglobin | | Haematocrit | | MCHC | |  | | | | | |
|  | *F_133_* | *P* | *F_117_* | *P* | *F_117_* | *P* |  |  |  |  |  |  |
| Acclimation_1_ | 0.24 | 0.6218 | 0.02 | 0.8708 | 1.22 | 0.2712 |  |  |  |  |  |  |
| Measurement_1_ | 0.24 | 0.6281 | 1.21 | 0.2736 | 0.20 | 0.6537 |  |  |  |  |  |  |
| Acclimation x Measurement_1_ | 0.67 | 0.4161 | 2.45 | 0.1205 | 7.72 | **0.0064** |  |  |  |  |  |  |

* ANCOVA was performed using log transformed independent variable and covariate (mass).

^ ANOVA was performed as there was no overall relationship with mass.

Table S3: Results of linear mixed effects models testing the effect of incubation treatment, acclimation treatment and acclimation duration (timepoint) on haematological parameters. Linear mixed effects models were created with tank as the random effect and P values computed using Kenward-Roger approximations with Type III SS. The highest order effects (P < 0.05) are in bold. Subscript values indicate group and residual degrees of freedom.

|  | Haematocrit | | Haemoglobin | | MCHC | |
| --- | --- | --- | --- | --- | --- | --- |
|  | F | P | F | P | F | P |
| Acclimation_1_ | 21.26 | 0.003 | 4.47 | 0.074 | 8.74 | 0.02 |
| Incubation_2_ | 0.71 | 0.51 | 2.15 | 0.16 | 1.25 | 0.31 |
| Timepoint_3_ | 12.27 | <0.0001 | 5.96 | <0.001 | 23.44 | <0.0001 |
| Acclimation x Timepoint_2_ | 11.83 | **<0.0001** | 38.35 | **<0.0001** | 17.79 | **<0.0001** |
| Incubation x Acclimation x Timepoint_12_ | 0.90 | 0.55 | 1.78 | 0.062 | 1.53 | 0.13 |
